# Integrative Study of the Crane Fly Genus *Brithura* Edwards, 1916 (Diptera: Tipulidae) in East Asia: First Larval Descriptions of the Genus and Insights from Adult Morphology and DNA Barcoding

**DOI:** 10.3390/insects16090978

**Published:** 2025-09-18

**Authors:** Virginija Podeniene, Sigitas Podenas, Dalius Butkauskas, Donatas Sneideris, Jin Whoa Yum, Neung-Ho Ahn, Soen Yi Kim, Jisoo Kim, Pavel Starkevich

**Affiliations:** 1 Institute of Biosciences, Life Sciences Centre of Vilnius University, Sauletekio Str. 7, LT-10257 Vilnius, Lithuania; sigitas.podenas@gamtc.lt; 2 State Scientific Research Institute Nature Research Centre, Akademijos Str. 2, LT-08412 Vilnius, Lithuania; dalius.butkauskas@gamtc.lt (D.B.); donatas.sneideris@gamtc.lt (D.S.); pavel.starkevic@gamtc.lt (P.S.); 3 Animal Resources Division, National Institute of Biological Resources, Incheon 22689, Republic of Korea; lestes93@korea.kr (J.W.Y.); paimon@korea.kr (N.-H.A.); lepi@korea.kr (S.Y.K.); 4 Biological Specimen Conservation Division, Nakdonggang National Institute of Biological Resources, Sangju 37242, Republic of Korea; j1soo@nnibr.re.kr

**Keywords:** China, integrative taxonomy, mtDNA, *COI*, Republic of Korea, larvae, adults

## Abstract

*Brithura* Edwards, 1916 (Diptera: Tipulidae) is a small genus of crane flies currently comprising 16 described species distributed across the East Palaearctic and Oriental regions. Although the adults of this genus rank among the largest representatives of the family Tipulidae, their immature stages have remained undocumented until now. In this study, mitochondrial cytochrome c oxidase subunit I (*COI*) gene fragment sequences (DNA barcodes) of *Brithura sancta* Alexander, 1929 were analyzed using both recently collected adult specimens from the Republic of Korea and historical museum specimens from China (collected in 1933). These sequences were compared with *COI* data obtained from larvae collected in Republic of Korea. We present the first description, with detailed illustrations and ecological information, of the previously unknown final instar larva of *Brithura*, specifically for the East Palaearctic species *B*. *sancta*. Diagnostic larval characters for the genus are discussed. Additionally, a redescription and comprehensive morphological documentation of the adult male and female *B*. *sancta*, including habitus and genitalia, are provided. This study represents the first phylogenetic contribution to the taxonomy of *Brithura* larvae based on mitochondrial *COI* sequence data.

## Simple Summary

The present study provides the first description of the final instar larva of *Brithura*, a genus of crane flies currently comprising 16 described species distributed across the East Palaearctic and Oriental regions. In addition, a redescription and detailed morphological documentation of the adult male and female of *B. sancta* are presented, alongside analysis of mitochondrial cytochrome c oxidase subunit I (*COI*) gene fragment sequences.

## 1. Introduction

The world fauna of the genus *Brithura* Edwards, 1916 currently contains 16 described species distributed throughout the East Palaearctic and Oriental regions [1]. A total of 12 species are recorded in China, and five species in India, sharing one common species, *B. imperfecta* (Brunetti, 1913) [1].

The genus was established by Edwards [2], with *Brithura conifrons* Edwards, 1916 designated as the type species by original designation. This species was later synonymized with *Brithura imperfecta* (Brunetti, 1913) [3].

Species of genus *Brithura* can be recognized by the following diagnostic characters: stout body; head with the vertex produced into a prominent tubercle; pleurotergal tubercle conspicuous, with dense pubescence on its dorsal face; dorsal sternopleurite often with hairs; costal border opposite stigma bulged in males of some species; and vein Sc1 retained in males and vein Rs short and strongly arcuated [2,4].

The family Tipulidae is the second-largest crane fly family, currently comprising 38 extant genera [1]. Larvae of this family occupy a wide range of ecological niches, including decaying wood, soil, leaf litter, organic detritus, moss, and both lentic and lotic aquatic habitats [5,6]. Despite the considerable body size of many species, some being the largest representatives of both Tipuloidea and Diptera, the larval stages of numerous taxa remain inadequately studied [7].

To date, larvae have been described for only 18 genera of Tipulidae [8] (Table 1). The state of knowledge remains markedly uneven across biogeographic regions: while larval morphology is relatively well documented for most genera occurring in Europe and North America, those from the Oriental Region, eastern Palaearctic, and especially the Southern Hemisphere remain poorly known, with many genera lacking any larval descriptions [7]. *Brithura* is one such genus, for which the immature stages and biological information were previously unknown.

DNA barcoding has introduced unprecedented opportunities in taxonomy, enabling the rapid and accurate association of larval stages with their corresponding adult species [21]. Recent advances in DNA extraction and PCR amplification techniques, including those applied to historical museum specimens, have further enhanced the utility of molecular tools in biodiversity research [22].

Historically, the majority of entomological studies have focused on adult insects, while larval stages have received comparatively little attention [23]. Due to the extended duration of insect life cycles, field collections often yield only a single developmental stage—most commonly adults—leaving a significant proportion of larval stages unassociated with identified adult taxa.

## 2. Materials and Methods

The adult material used in this paper was obtained from the United States National Museum (USNM), Smithsonian Institution, Washington DC, USA; the National Institute of Biological Resources (NIBR), Incheon; and the Korea University (KU), Seoul, Republic of Korea.

Adult crane flies were collected by insect net. Some specimens were preserved dry in envelopes in the field and later mounted in the laboratory on their side on a paper point with legs generally surrounding the insect pin. The specimens are pinned except when noted otherwise. Larvae were hand-collected by V. Podeniene in the Republic of Korea in 2022.

Adult specimens were studied with a Nikon SMZ800 stereomicroscope (Nikon, Tokyo, Japan). Photographs of adult specimens were taken with a Canon EOS 6D camera and Canon MP-E65 mm macro lens (Canon, Tokyo, Japan) using a MJKZZ automated focus stacking rail set (MJKZZ.de, Vienna, Austria). Pictures of male and female terminalia were taken with an INFINITY-1 camera (Teledyne, Thousand Oaks, CA, USA) mounted on a Nikon Eclipse Ci-L stereomicroscope (Nikon, Tokyo, Japan) and with a Canon EOS 80D camera (Canon, Tokyo, Japan) mounted on an Olympus SZX10 dissecting microscope (Olympus, Tokyo, Japan). The obtained image layers were stacked using the program ZereneStacker 1.04 and edited with Photoshop 26.5.0.

Potassium hydroxide was used to macerate dissected parts of adult insects and larvae [24]. Genitalia were studied after heating them in 10% KOH solution for 10–15 min and then preserved in microvials filled with glycerol and attached to the pin with the corresponding specimen. Redescription and illustrations are based on Korean and Chinese material.

Larvae are preserved in 70% ethanol. The larval head capsules were cleaned in a hot, 10% KOH solution for several hours, and temporary slides were made in gelatin glycerol. The spiracular fields of the larvae were excised, and temporary slides were made in gelatin glycerol. General photographs of larvae, pupae, and head capsules were made with a Canon EOS 80D digital camera using a Canon MP-E65 mm macro lens. All larval material is stored in the NIBR collection.

Molecular analysis was used to associate specimens of *B. sancta* from the Republic of Korea and China. A phylogenetic tree was based on molecular sequence data from cytochrome c oxidase subunit I (*COI*). DNA extraction was performed using GeneJet Genomic DNA Purification Kit (Thermo Fisher Scientific Baltics, Vilnius, Lithuania) according to the manufacturer’s instructions. The DNA samples were kept frozen at −20 °C. PCR amplification of *COI* gene sequence was performed using CLepFolF (5′-ATTCAACCAATCATAAAGATATTGG-3′) and CLepFolR (5′-TAAACTTCTGGATGTCCAAAAAATCA-3′) primer pairs [25]. PCR reactions were performed using DreamTaq PCR Master Mix (Thermo Fisher Scientific Baltics, Vilnius, Lithuania) according to the manufacturer’s instructions. The PCR cycling conditions were the following: initial denaturation for 5 min at 95 °C, 35 cycles of 45 s at 95 °C, 45 s at 54 °C, 60 s at 72 °C, and final extension for 10 min at 72 °C. PCR products were observed in agarose gel and purified using Exonuclease I and FastAP Thermosensitive Alkaline Phosphatase (Thermo Fisher Scientific Baltics). All samples were sequenced directly with the 3500 Genetic Analyzer (Applied Biosystems, Foster City, CA, USA) using the same forward and reverse primers as for PCR.

Molecular analysis was used to associate adult individuals with larvae. Total genomic DNA was extracted from the middle body segments of larva using a QIAamp DNA Micro Kit (Qiagen, Hilden, Germany). Standard PCR amplification and sequencing protocols were used to generate *COI* fragment sequences [9,10]. The target fragment of *COI* was amplified in 20 µL reactions containing AccuPower PCR PreMix (Bioneer Co., Daejeon, Republic of Korea), 1 U Top DNA polymerase, dNTPs (10 mM), Tris-HCl (pH 9.0), KCl (30 mM), MgCl2 (1.5 mM), 3 µL (5–50 ng) template DNA, and 1 µL of each primer (LCO1490 and HCO2198; 10 pM each) [11]. Amplification was performed using the following thermal cycling program: 94 °C for 4 min; 35 cycles of 94 °C for 0.5 min; 48 °C for 0.5 min; 72 °C for 1 min; and a final extension at 72 °C for 10 min. PCR products were sequenced by Macrogen Inc. (Seoul, Republic of Korea).

*Brithura sancta COI* sequences were submitted to GenBank (Table 2). The molecular analyses were performed using the MEGA11 program [26] to produce a maximum likelihood tree [27] and subjected to a bootstrap test with 1000 replicates. While obtaining the maximum likelihood tree, we used *COI* sequences of corresponding length deposited by other authors in the BOLD Systems data portal (https://portal.boldsystems.org, accessed on 11 June 2025) and GenBank (https://www.ncbi.nlm.nih.gov/genbank, accessed on 11 June 2025) (Table 2).

Descriptive terminology of adults generally follows that of Cumming and Wood [28]. The term ventral lobe of appendage of sternite 9 (=fragmentum [29], =A9s [30]) is adopted from Gelhaus [31]; the term gonocoxal fragment (=sclerites sp1 and sp2 [32], =genital bridge [33]) for inner structure covered by epandrium is adopted from Brodo [34]. Descriptive terminology of larva generally follows that of Gelhaus [35] and Neugart et al. [36].

## 3. Results


***Brithura sancta* Alexander, 1929**
Figure 1, Figure 2, Figure 3, Figure 4, Figure 5, Figure 6, Figure 7, Figure 8, Figure 9, Figure 10, Figure 11 and Figure 12

*Brithura sancta* Alexander, 1929: 317 (original description) [37]; Savchenko, 1983: 524 [38]; Oosterbroek and Theowald, 1992: 58 [39]; Yi, 2024 [40]

*Tipula* (*Brithura*) *sancta* Edwards 1932: 240 [41]; Alexander, 1935: 88 [4].

 

**Type of material examined. CHINA** • Holotype ♂; Ton Chessu, a temple in hills west of Peking; 27 August 1921; A. P. Jacot; USNM.

**Additional material examined, adult specimens. CHINA** • 1 ♀; [Sichuan] Szechwan; BehLuhDin, 30 mi N. Chengtu [N. Chengdu]; alt. 6000 ft.; VIII-13 1933; D.C. Graham; Det. C.P.Alexander Metat 1934; USNM • 1 ♂ 2 ♀♀; same data as for preceding; VIII-14 1933; USNM • 1 ♂ 2 ♀♀; same data as for preceding; VIII-16 1933; USNM • 1 ♀; same data as for preceding; VIII-18 1933; USNM • 1 ♂; same data as for preceding; VIII-23-24 1933; terminalia dissected; 01843358; USNM • 1 ♂; same data as for preceding; terminalia dissected; 01843359; GenBank Accession number OQ368740; USNM • 1 ♂ 1 ♀; same data as for preceeding; VIII-24 1933; 01843360; 01843361; terminalia dissected; USNM • 1 ♂ 1 ♀; [Sichuan] Szechwan; Kuanshien; 3200 ft.; vii-18-20-1933; D.C. Graham; Det. C.P.Alexander Metat 1934; USNM • 1 ♀; [Sichuan] Szechwan; WeiChow, 65 mi N. Chengtu; 5000–8000 ft.; vii-25-1933; D.C. Graham; Det. C.P.Alexander Metat 1934; USNM. **REPUBLIC OF KOREA** • 1 ♂; Gyeongsangbuk-do; Yeongju, Dansan-myeon, Dangok-ri; N36.96611, E128.57195; 27 June 2013, coll. S. Kim; NIBR; • 1 ♂; Gangwon-do; Chuncheon-si, Dongsan-myeon, Bongmyeong-ri; KNU Experimental Forest; N37.78194, E127.81973; alt. 197 m; 9 July 2015, coll. S. Kim; GenBank Accession number OQ368738; NIBR • 1 ♀; same data as for preceding; NIBR • 1 ♀; same data as for preceding; GenBank Accession number OQ368739; NIBR • 1 ♀; Gangwon-do; Pyeongchang-gun; Mt. Odaesan; [37°44′25”N 128°35′02”E]; 24. VI. 1994; leg. Hyeon-Ju Oh; KU.

**Additional material examined, immature stage. REPUBLIC OF KOREA** • 11 larvae; Gangwon-do, Yeongwol-gun, Yeongwol-eup, Heungwol-ri; N37.1099210, E128.4658514; 17.VI.2022; leg. V. Podeniene; NIBR; one larva dissected with GenBank Accession number PV834789.

**Diagnosis.** *Brithura sancta* can be recognized by its large, stout body with a clove-brown coloration, the head bearing a prominent tubercle, and femora marked with a pale preapical ring. Males are further characterized by the hypandrium produced into distinct tubercle and a complex outer gonostylus, which posteriorly bears an elongated, curved, stick-shaped lobe. Females can be identified by the extended posterolateral angles of tergite 10.

**Figure 1:**
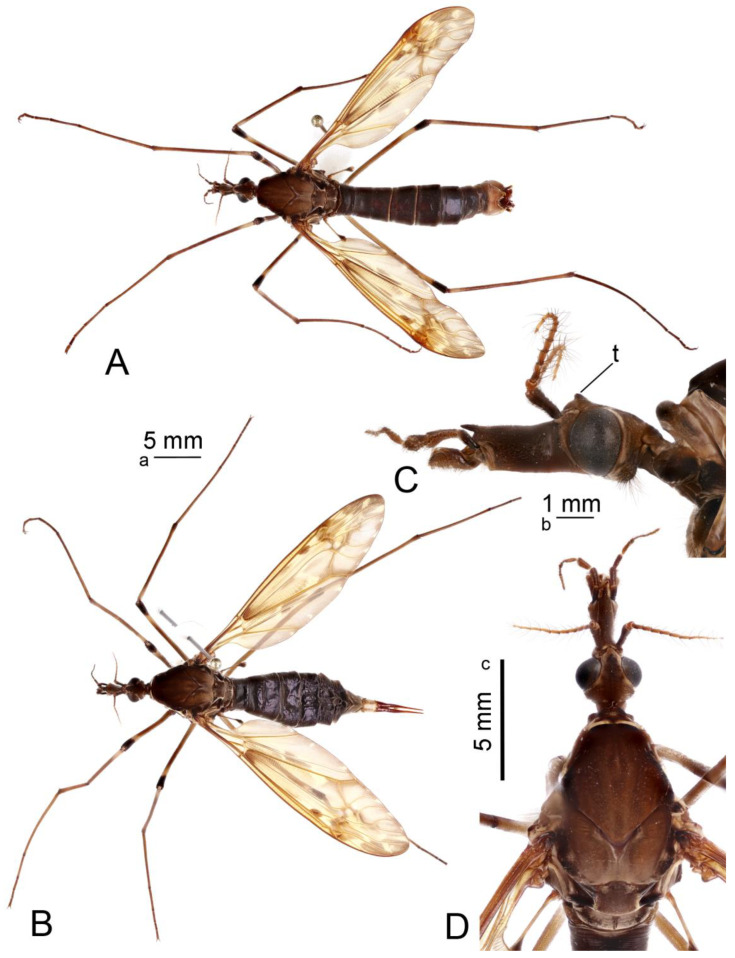
Habitus of adults *Brithura sancta* (Republic of Korea). (**A**) Male, dorsal view; (**B**) female, lateral view; (**C**) male head, lateral view; (**D**) male head and thorax, dorsal view. Scale a: (**A**,**B**); b: (**C**); c: (**D**). Abbreviation: t, tubercle.

**Redescription. Adult male.** Body length 27.1–32.2 mm, wing length 21.1–24.1 mm, antenna length 4.3–5.5 mm (N = 8). General body coloration dark brown (Figure 1A,B,D).

*Head*. Head brown, vertex with distinct, darker brown tubercle (Figure 1C,D). Rostrum brown, nasus distinct, darker brown. Palpus with basal segment dark brown, remaining segments brown. Antenna 13-segmented, when bent backward, reaching the presutural scutum. Scape brown, pedicel and flagellum brownish yellow (Figure 1C). Each flagellomere has a small basal enlargement except the first. Apical flagellomere small. Longest verticils are longer than the corresponding segments. Scutellum brown, with a darker median line.

*Thorax*. Pronotum brown, with anterolateral margins dark brown (Figure 1D). Prescutum and presutural scutum clove-brown with lateral margins darkened. Median pair of stripes reddish brown, indistinctly bordered by brown, fused anteriorly and posteriorly, separated in the middle. Lateral stripes indistinct. Postsutural scutum medially gray brown, each lobe with two clove-brown spots, bordered by brown. Mediotergite brown, dusted with gray, with brown median line. Pleuron clove-brown. Legs with coxae and trochanter brown. Femora brown, blackish brown tip preceded by pale subterminal ring (Figure 1A,B). Tibia basally pale, getting brown towards the dark brown tip. Tarsal segments brown. Claw with the tooth. Wing with ground color yellow (Figure 1A). Costal region opposite to the stigma dilated, dark pattern at bases of cells r, m, origin of Rs and area covering stigma and cord. Outer ends of cells from R_3_ to A_1_ clouded, each with small yellow spots. Vein Sc_1_ present, Rs strongly arcuate.

*Abdomen*. Abdominal tergites blackish brown, epandrium cinnamon brown (Figure 1A). Sternites reddish brown.

**Figure 2:**
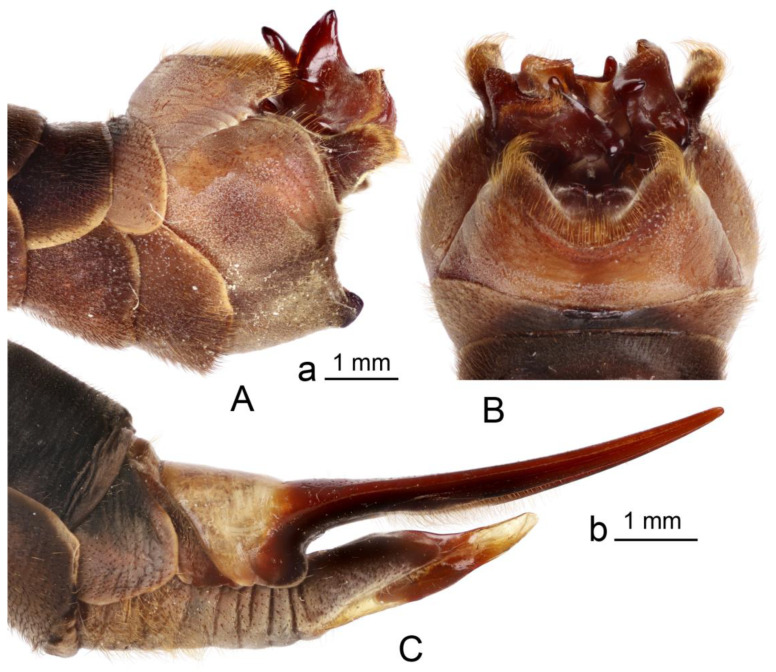
*Brithura sancta*, male and female terminalia (Republic of Korea). (**A**) Male hypopygium, lateral view; (**B**) male hypopygium, dorsal view; (**C**) female ovipositor, lateral view. Scale a: (**A**,**B**); b: (**C**).

**Figure 3:**
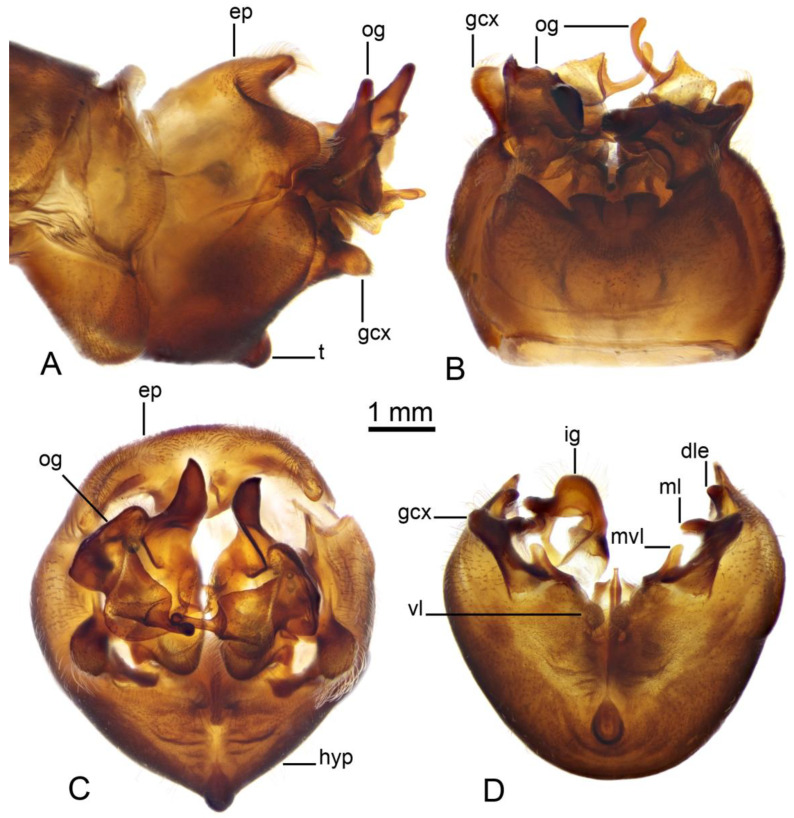
Male terminalia of *Brithura sancta* (China). (**A**) Hypopygium, lateral view; (**B**) hypopygium, dorsal view; (**C**) hypopygium, caudal view; (**D**) hypopygium, caudal view, epandrium, outer gonostyli and right inner gonostylus removed. Abbreviations: dle, dorsolateral extension of lateral sclerite of gonocoxal fragment; ep, epandrium; gcx, gonocoxite; ig, inner gonostylus; hyp, hypandrium; t, tubercle of sternite 9; ml, mesal lobe of lateral sclerite of gonocoxal fragment; mvl, mesoventral lobe of lateral sclerite of gonocoxal fragment; og, outer gonostylus; vl, ventral lobe of A9s.

**Figure 4:**
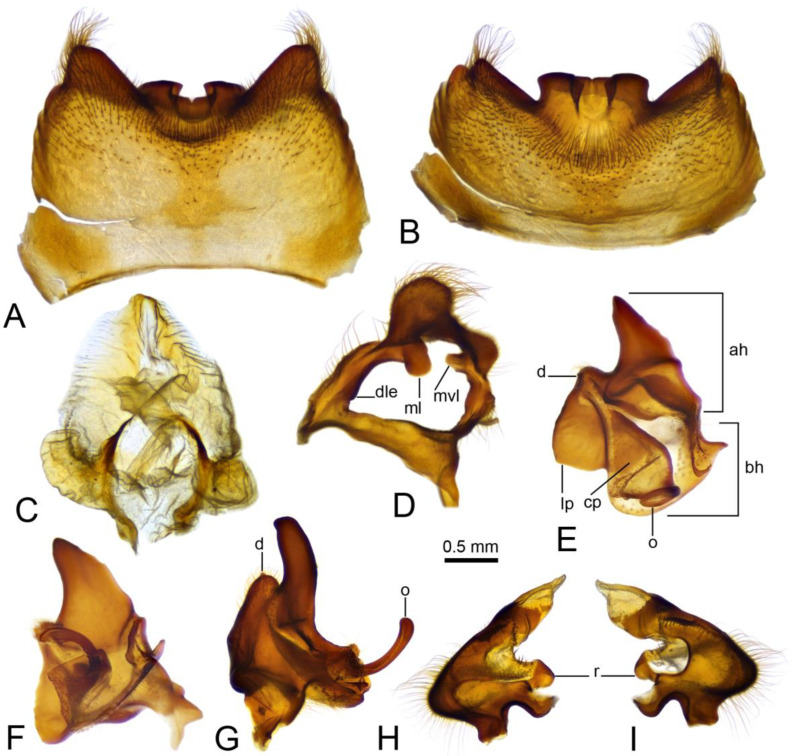
Male terminalia of *Brithura sancta* (China), epandrium, proctiger, gonocoxite, lateral sclerite of gonocoxal fragment, outer and inner gonostyli. (**A**) Epandrium, dorsal view; (**B**) epandrium, caudal view; (**C**) proctiger; (**D**) right gonocoxite and lateral sclerite of gonocoxal fragment; (**E**) left outer gonostylus, inner view; (**F**) left outer gonostylus, inner view at slightly different angle; (**G**) right outer gonostylus, outer view; (**H**) left outer gonostylus, inner view; (**I**) left outer gonostylus, outer view. Abbreviations: ah, apical half of the main body of outer gonostylus; bh, basal half of the main body of outer gonostylus; cp, caudal part of outer gonostylus; d, denticle; dle, dorsolateral extension of lateral sclerite of gonocoxal fragment; lp, lateral part of outer gonostylus; ml, of lateral sclerite of gonocoxal fragment; mvl, mesoventral lobe of lateral sclerite of gonocoxal fragment; o, outgrowth; r, ring.

**Figure 5:**
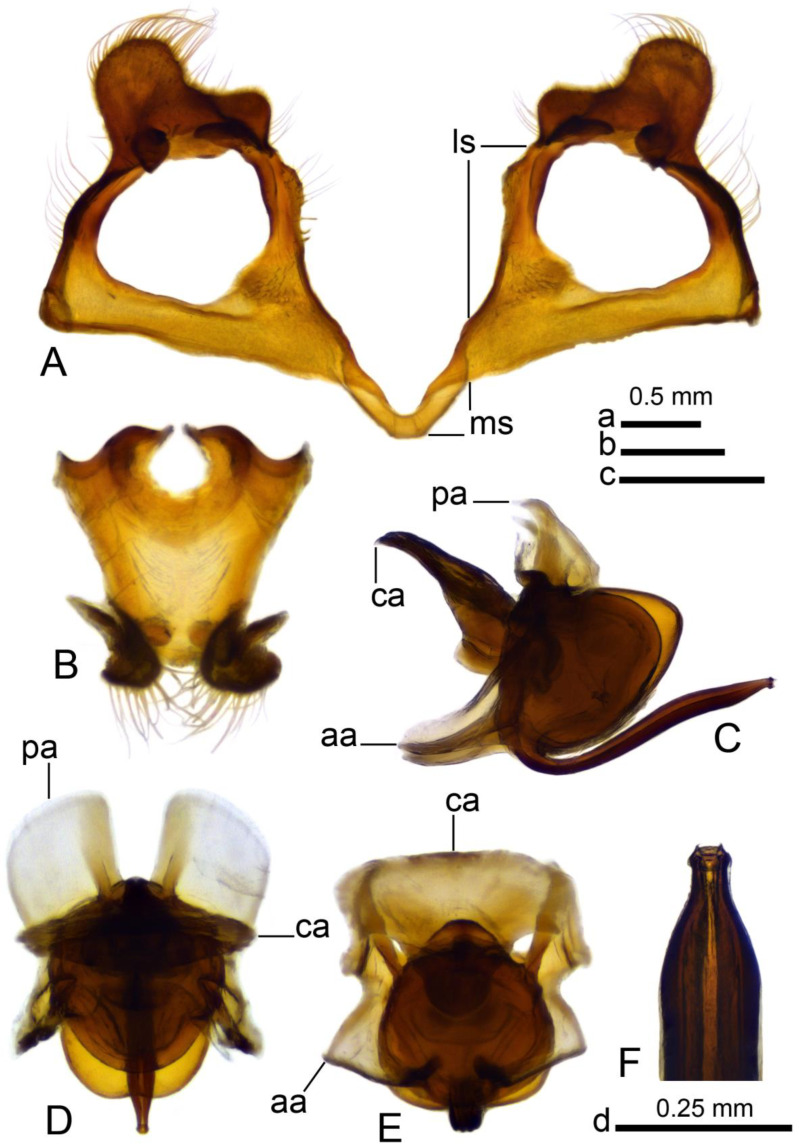
Male terminalia of *Brithura sancta* (China), gonocoxites with gonocoxal fragment, adminiculum and sperm pump (China). (**A**) Gonocoxites and gonocoxal fragment; (**B**) adminiculum, ventral view; (**C**) sperm pump, lateral view; (**D**) sperm pump, ventral view; (**E**) sperm pump, ventral view at different angle; (**F**) apical part of intromittent organ. Abbreviations: aia, anterior immovable apodeme; ca, compressor apodeme; ls, lateral sclerite of gonocoxal fragment; ms, medial sclerite of gonocoxal fragment; pia, posterior immovable apodeme. Scale a: (**A**); b: (**C**–**E**); c: (**B**); d: (**F**).

*Hypopygium* (Figure 2A,B and Figure 3, Figure 4 and Figure 5). Epandrium and hypandrium fused at base (Figure 2A and Figure 3A). Epandrium relatively large, in the shape of nearly rectangular plate, posterior portion covered with setae except posteromedian extension (Figure 2B, Figure 3B and Figure 4B). Posterior margin with median U-shaped emargination. Posterolateral angles darkened, produced into obtuse lobes. Posteromedian extension nearly rectangular, darkened, possesing median groove. Proctiger partly sclerotized, basally with pair of sac-shaped formations and narrow blackened sclerites, presumably representing cerci (Figure 4C). Gonocoxite narrow, apically producing into flattened, rounded lobe covered with setae and smaller, ventromedian lobe (Figure 2B, Figure 3B–D, Figure 4D and Figure 5A). Mesal surface of gonocoxite extended into additional mesal lobe (Figure 4D). Outer gonostylus complicated (Figure 3B,C and Figure 4E–G), and may be subdivided into three basic parts: the main body, caudal part, and lateral part (Figure 4E). The apical half of the main body sclerotised, keel-shaped, its basal margin slightly elevated. Basal half of the main body yellow, rounded, mesal margin laterally extended into a short, narrowed lobe. Caudal part of outer gonostylus is produced from basal half of the main body as additional fold, detached laterally by the pale suture and completely separated in the middle. The caudal part has the shape of a nearly rectangular lobe, its mesal margin is produced into a remarkable outgrowth. The outgrowth elongated and curved, with the apical part lightly swollen. The lateral part of the outer gonostylus is laterally attached to the main body. The lateral part flattened, basally dilated, distal end rounded, with an additional short ridge terminating in an obtuse denticle. Inner gonostylus with apical portion flattened, terminating in an obtuse pale beak (Figure 3B,D and Figure 4H,I) Basal portion rounded, covered with setae. Posterior condyle flattened, prominent, anterior condyle nearly rounded. Inner margin with broad round incision, its basal angle (above anterior condyle) producing into elongated extension which is fused with medially extended, mesal part of the sclerite forming closed ring (Figure 4H,I). The inner margin above the fusion creasy, covered with short setae. Gonocoxal fragment with medial sclerites fused at base, small, V-shaped (Figure 3D, Figure 4D and Figure 5A). Lateral sclerites large, hypertrophied, basally flattened, fused with gonocoxite dorsolaterally and mesoventrally. Mesoventral portion terminating into free, short, mesal and mesoventral lobes, dorsolateral portion with additional, short, dorsolateral extension (Figure 3D and Figure 4D). Hypandrium large, ventrally produced into a rounded tubercle (Figure 2A and Figure 3A,B,D). Ventral lobe of A9s in the shape of a rounded sclerite, the surface covered with setae (Figure 3D). Adminiculum flattened, covered by hypandrium and inner gonostyli (Figure 3D). Distal portion with nearly rectangular angles (Figure 5B). Sperm pump swollen, caudal part extended and narrowed (Figure 5C–E). Compressor apodeme and anterior immovable apodeme flattened, anterior apodeme flattened, laterally narrowed. Intromittent organ stout, relatively short, shorter than the sperm pump (Figure 5C,F). Apical part narrowed (Figure 5F).

**Adult female.** Body length 29.1–44.0 mm, wing length 24.3–29.0 mm, antenna length 4.3–5.9 mm (N = 12). Generally similar to male by body coloration (Figure 1B).

**Figure 6:**
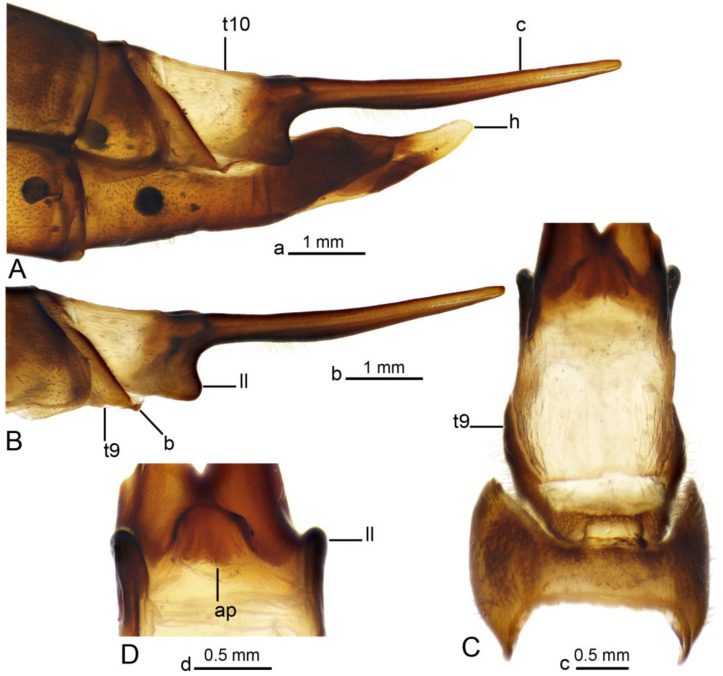
Female terminalia of *Brithura sancta* (China). (**A**) Ovipositor, lateral view; (**B**) terminal tergites and cercus, lateral view; (**C**) tergites 8–10, dorsal view; (**D**) anal plate, ventral view. Abbreviations: ap, anal plate; b, band (its fragment); c, cercus; h, hypovalva; ll, lateral lobe of t10; t10, tergite 10; t9, tergite 9. Scale a: (**A**); b: (**B**); c: (**C**); d: (**D**).

**Figure 7:**
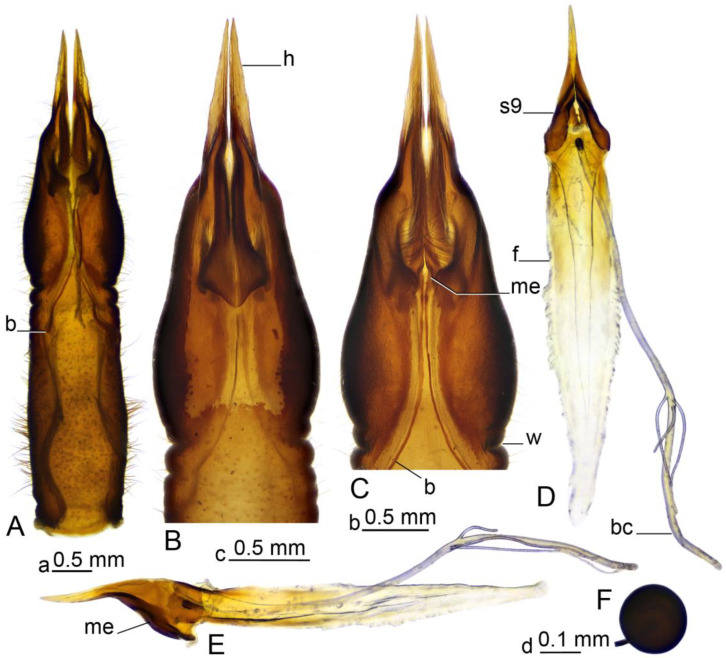
Female terminalia of *Brithura sancta* (China). (**A**) Sternite 8, dorsal view; (**B**) anterior part of sternite 8, ventral view; (**B**) anterior part of sternite 8, dorsal view; (**D**) sternite 9, furca and bursa copulatrix, dorsal view; (**E**) sternite 9, furca and bursa copulatrix, lateral view; (**F**) spermatheca. Abbreviations: b, band; bc, bursa copulatrix; f, furca; h, hypovalva; me, mesal edge of sternite 9 (band’s attachment point); s9, sternite 9; w, wrinkle. Scale a: (**A**); b: (**B**,**C**); c: (**D**,**E**); d: (**F**).

*Female terminalia* (Figure 2C, Figure 6 and Figure 7). Tergite 9 partly reduced (Figure 6C), lateral part distally acute (Figure 6A). Tergite 10 pale (Figure 2C and Figure 6A–C), lateral angles extended into blackened lobes (Figure 6B). Cercus nearly straight, slender, and elongated (Figure 2C and Figure 6A,B). Anal plate posteriorly and laterally bordered by elevated margins (Figure 6D). Sternite 8 with posterior half separated from the rest of the body by distinct wrinkles (Figure 2C and Figure 7A–C). Hypovalva pale yellow, nearly blade-shaped (Figure 7A–C). The base of hypovalva extended into longer, sclerotized, anteriorly flattened and dilated plate (Figure 7B). Sternite 8 forming nearly rounded concavity near the base of cerci and apex of sternite 9, its surface covered by microscopic filaments and long setae (Figure 7C). Sternite 9 apically elongated into narrow extension, lateral parts flattened, basally dilated (Figure 7E). A pair of narrow bands starts at the mesal edges of sternite 9 (Figure 7A,C). The band fused with the inner margin of sternite 8 (Figure 7C,E) and reaching the lateral end of tergite 9 (Figure 6B). Furca flattened, tongue-shaped, fused with sternite nine ventrally and laterally, about 2.6 times as long as sternite 9 (Figure 7D,E). Bursa copulatrix elongated, its aperture with rounded sclerotization (Figure 7D,E). Three spermatheca are present, spherical in shape (Figure 7F).

**Figure 8:**
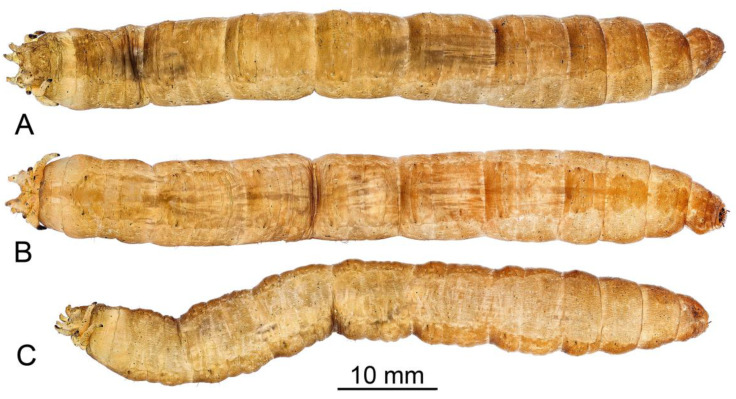
General view of the larva of *Brithura sancta* (Republic of Korea). (**A**) Dorsal view; (**B**) ventral view; (**C**) lateral view.

**Last instar larva**. Length 28–68 mm, width 7–9 mm (N = 11). Body light brown, covered with short light brown hairs; dorsal and ventral sides darker than lateral sides (Figure 8).

**Figure 9:**
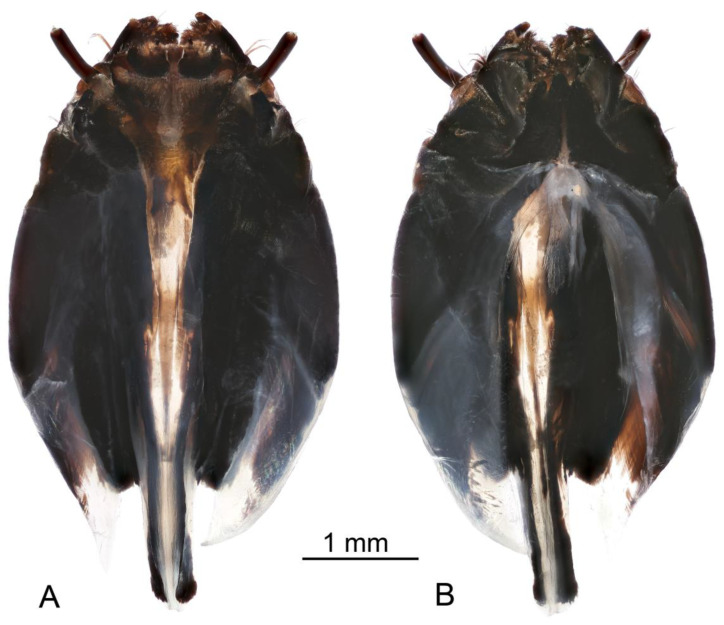
Larva of *Brithura sancta*, general view of head capsule (Republic of Korea). (**A**) Dorsal view, (**B**) ventral view.

**Figure 10:**
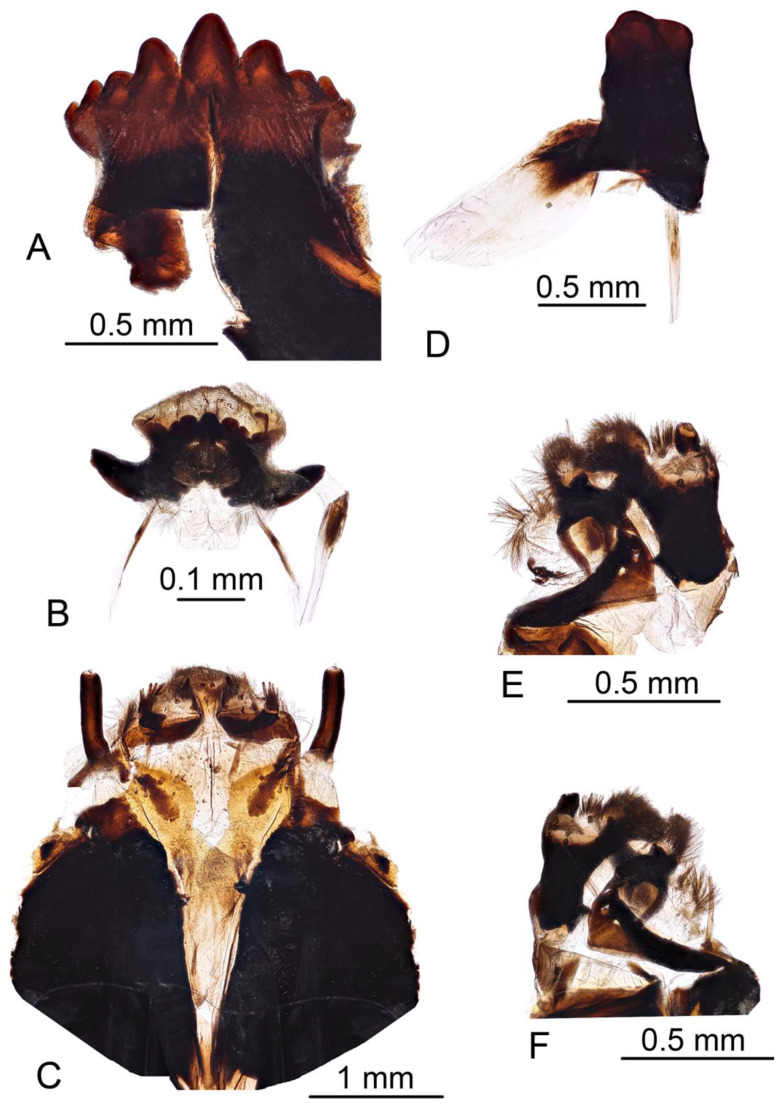
Larva of *Brithura sancta*, head capsule (Republic of Korea). (**A**) Hypostomium; (**B**) prementum; (**C**) frontoclypeus, labrum and antenna; (**D**) left mandible; (**E**) left maxilla, dorsal view; (**F**) left maxilla ventral view.

*Head capsule* (Figure 9 and Figure 10). Length 2.3 mm, width 1.2 mm. Head capsule prognathous, hemicephalic, oval in shape, slightly depressed dorsoventrally and heavily sclerotized (Figure 9). Internolateralia and externolateralia separated by incisions that reach about one third of head capsule length. The externolateralia widely separated ventrally (Figure 9B). Premaxillary suture separates side plate from the rest of the head capsule. Side plate wide and elongated, with two sensory pits and two long setae anteromedially, a short seta located posteromedially (Figure 9B). Hypostomium symmetrical, basally fused with the ventral margins of genae and side plates. It bears nine teeth: three prominent teeth in the middle and three much smaller teeth on both sides (Figure 10A). Prementum visible from below, it bears five small blunt teeth on the anterior margin, sides strongly sclerotized (Figure 10B). Labial area entirely covered with firm bristles and bearing a pair of cone-shaped palpes. Prementum dorsally fused with hemispherical and membranous hypopharynx which is covered with numerous short hairs. Lateral arm of hypopharynx curved and strongly sclerotized. Frontoclypeus fused with internolateralia. Clypeal part of frontoclypeus membranous, frontal part sclerotized. One long seta and a sensory pit located on the anterior part of clypeus, one long seta and four sensory pits located near the inner margin of antenna (Figure 10C). The clypeolabral suture obscure. The dorsal ecdysial sutures (frontal sutures) present, they meet each other posteriorly and form a short median coronal suture. Ecdysial sutures enclose the V-shaped frontoclypeus and extend only to the base of the clypeus anteriorly. Labrum trapezoidal in shape and composed of two triangular plates separated by membranous area (Figure 10C). Apical part of labrum and epipharynx covered with numerous short hairs. Membranous part of labrum with a pair of medium-long setae in the middle. Labral plates widely sclerotized posteriorly and bears numerous long firm spines on outer margin. Each plate bears one long and one medium length papillae on the anterior part, one long flattened seta almost in the middle, and a sensory pit on the posterolateral part (Figure 10C). Antenna elongated, cylindrical. It has just one cylindrical segment, which is almost four times as long as width at the base (Figure 10C). Apically it has one small cone-shaped and several (exact number is difficult to establish) small peg-like sensillae, dorsally it has a sensory pit near the middle. Mandible one segmented and more sclerotized than the rest of the head capsule, with two blunt inconspicuous teeth (Figure 10D). Both teeth are similar in size and shape. Prostheca or lacinia mobilis present on the dorsal side of mesal mandibular base. It is sclerotized, distinctly widening distally, and set with numerous hairs. Lateral margin of mandible has one very short seta near the base. Mandibles operate in horizontal plane. Conspicuous larval eye spot present below the base of mandible. Maxilla consists of cardo, outer and inner lobes. Cardo wedge-shaped, anterior part heavily sclerotized. It bears two long flattened setae and a sensory pit near the distal end; a long seta present close to its base (Figure 10E,F). Outer lobe (stipes) sclerotized except apex, which bears prominent cylindrical palpus with several sensory structures. Three short sensory structures and several long sclerotized spines (exact number is difficult to establish) located on inner margin of stipes. Inner lobe (galea fused with lacinia) ventrally bears elongated narrow sclerite extending around inner margin onto its dorsal surface; diamond-shaped sclerite present dorsally at the base (Figure 10E); apical part with numerous short hairs, a long setae and prominent sensory structure. Lacinia armed with several stout bristles.

*Thorax*. All thoracic segments wider than long (Figure 8).

**Figure 11:**
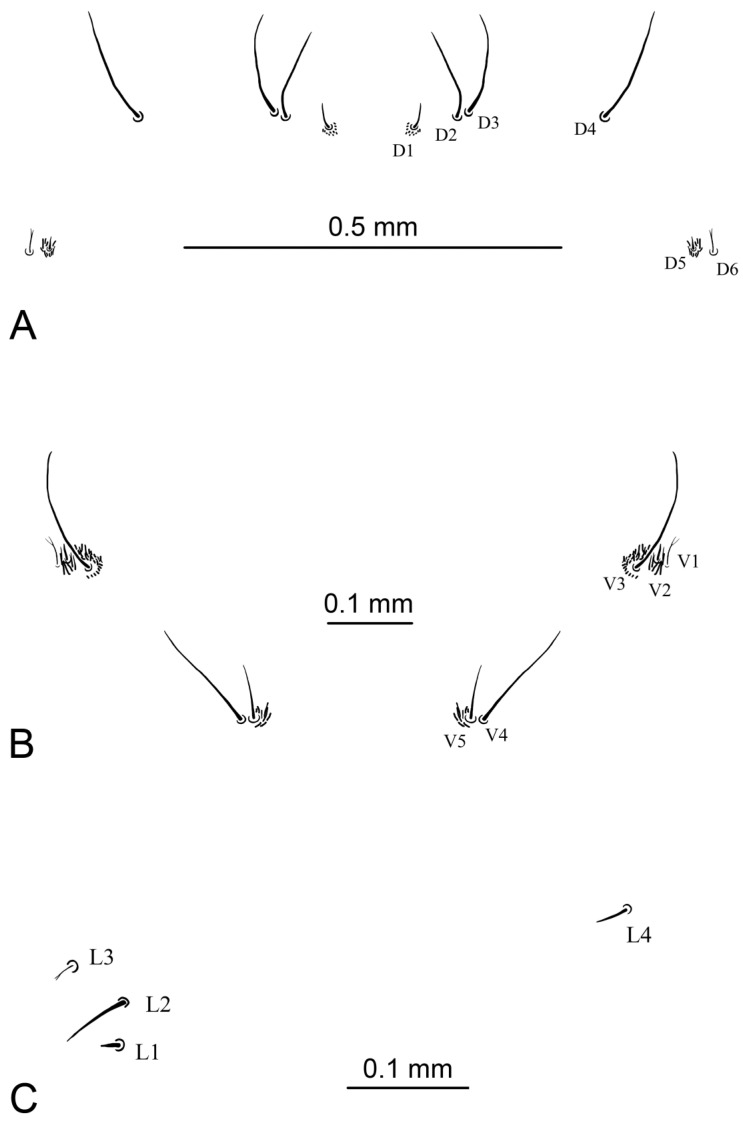
Abdominal setae of larva of *Brithura sancta* (Republic of Korea). (**A**) Dorsal abdominal setae; (**B**) ventral abdominal setae; (**C**) lateral abdominal setae.

*Abdomen*. First abdominal segment almost twice as wide as long. Abdominal segments II–VII almost as long as wide. Most of macrosetae dark brown except D6, V1, and L3. Dorsal setae D2–D4 are the longest (Figure 11A), seta D1 half length D2–D4, seta D5 very short and surrounded by longer dark hairs, two times shorter than D1. Seta D6 thin, pale, and branched; slightly shorter than D1. Setae D2 with D3 and D5 with D6 are close to each other and separate from the others. Ventral setae V3 and V4 are the longest setae (Figure 11B). V5 is half length of V3–V4. Seta V2 is very short and surrounded by long dark hairs, three times shorter than V5. Seta V1 thin, pale, and branched; half length of V5. Setae V5 with V4 and V3–V1 are close to each other and separate from the others. Lateral seta L3 thin, pale, and branched (Figure 11C). Seta L2 the longest and situated dorsolaterally to L1. L1 and L3 almost equal in length and are three times shorter than L2. L4 is half length of L2.

**Figure 12:**
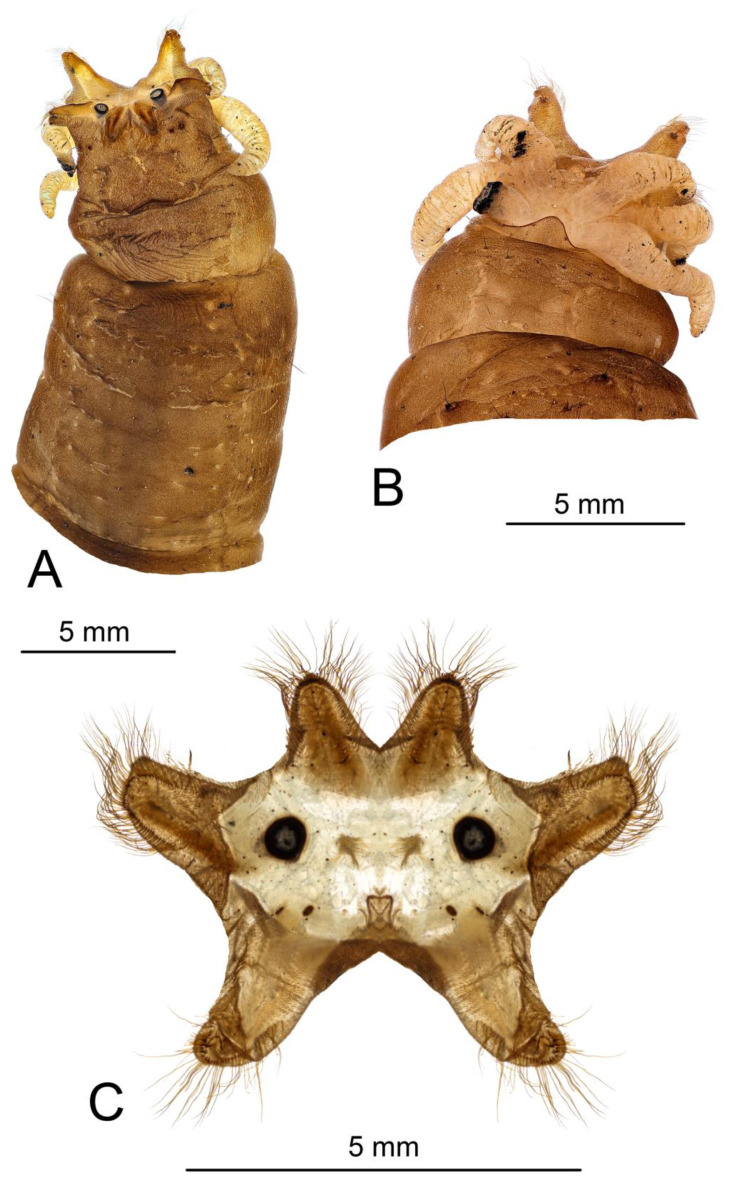
Larva of *Brithura sancta* (Republic of Korea). (**A**) Terminal segment dorsal view; (**B**) terminal segment ventral view; (**C**) spiracular field, caudal view.

*Terminal segment* (Figure 12). All spiracular lobes conical, elongated, and narrow. Dorsal and lateral lobes almost 1.5 times as long as the width at base, ventral lobes twice as long as wide. Lateral lobes equidistant from dorsal and ventral lobes. Lateral lobes only slightly longer than dorsal lobes, ventral lobes almost twice as long as dorsal lobes. Apical half of lobes with well-developed border of setae, longest setae twice the basal width of the lobe. The basal part of the lobes and the area between the lobes not bordered with setae (Figure 12). The posterior surface of the spiracular field not covered with short microscopic hairs, and the spiracular lobes are able to close. Apical part of lateral and ventral spiracular lobes with small outgroves on the anterior surface, each outgrove bears several long setae. Dorsal lobe of spiracular field entirely covered with brown sclerite, margins of sclerite darker than the middle part. Inner margin of lateral lobe with narrow, dark, elongated sclerite from base to apex. Two small black spots near the base of each ventral lobe. Ventral lobe entirely covered with very light brown sclerite. Spiracle circular, black, distance between spiracles more than four diameters of a spiracle (Figure 12C). Six white and elongated anal papillae arranged into lateral and median pairs, lateral pair single, median papillae paired (Figure 12B). Marginal band of anal field dark brown.

**Habitat.** Larvae were found in sandy mud under stones and leaf litter in a shallow, steep stream together with larvae of *T.* (*Nippotipula*) Matsumura, 1916.

**Distribution**. China [1] and Republic of Korea [40].

***COI* sequencing.** The partial, 620 bp length, *COI* gene nucleotide sequences were obtained from three Korean (two adult (OQ368738, OQ368739) and one larva (PV834789)) and one very old Chinese adult (OQ368740) specimens. The latter was identified as *B. sancta* by the author who deposited it into collection of the museum in 1933. All three sequences isolated from Korean *B. sancta* specimens were identical and had 96.5% nucleotide sequence similarity to the old *B. sancta* isolate from China OQ368740. They also shared 99.2-100% identity with six isolates from S. Korea (ON732830-ON732835) and -93.3-93.6% similarity to five isolates of morphologically different *Brithura* species from China [Taiwan] (CMNH544017, CMNH544493, CMNH544500, CMNH551721, CMNH575752). When comparing to other genera, Korean isolates shared 91.4% identity with *Tipula (Nippotipula) coquilletti* (ON732843), 90.9% with *Tipula (Tipula) mediterranea* (MZ196738), and 89.2% with *Tipula (Nobilotipula) collaris* (CMNH475020).

## 4. Discussion

**Figure 13:**
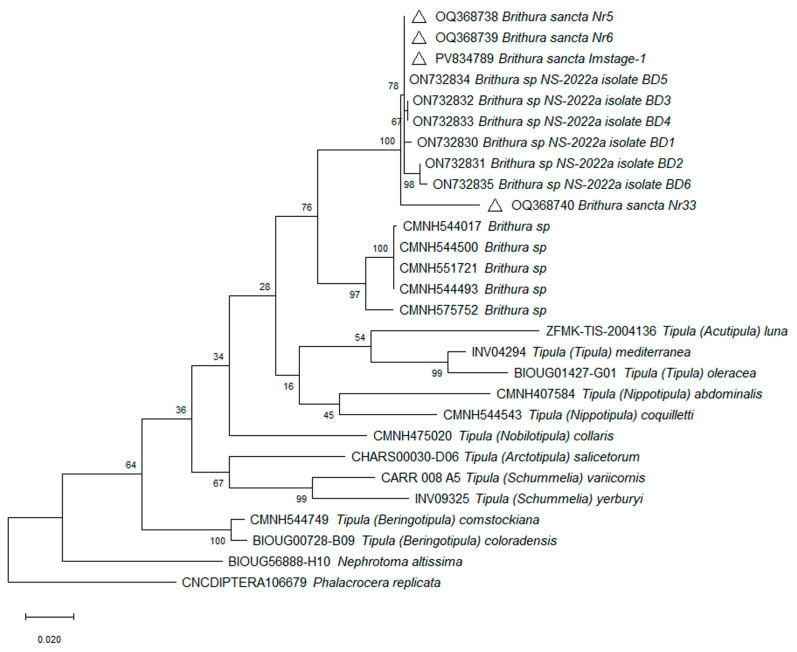
Phylogenetic tree based on cytochrome oxidase subunit I gene sequence. Constructed using the Maximum Likelihood method based on the Tamura–Nei model [13] and subjected to a bootstrap test with 1000 replicates. The scale bar represents the number of base substitutions per site. Triangles mark the isolates obtained in this study.

*Brithura sancta* is distinguished by a remarkable three-lobed outer gonostylus. The most closely related species is *B. triprocessa* Men and Liu, 2019 [42] in a similar shape of outer gonostylus consisting of three parts, still having different shapes (ref. [42]: Figures 13–17). The main body of the outer gonostylus in *B*. *triprocessa* terminates in an obtuse lobe, while the caudal part has the shape of a sharp process separated by a light region. The lateral part of the outer gonostylus (=inner obtuse lobe [42]) is generally similar to that of *B. sancta*, but lacks denticle.

*Brithura sancta* is also characterized by an inner gonostylus having a specific ring and a pair of long bands which are fused with the mesal edges of sternites 9, along the margin of sternite 8, and reaching the distal ends of tergite 9.

Male of *B. sancta* lacks the distal lobe of A9s, a structure common, e.g., in *T.* (*Eremotipula*) Alexander, 1965, *T*. (*Lunatipula*) Edwards, 1931, and *T*. (*Vestiplex*) Bezzi, 1924. The part of *T*. (*Vestiplex*) species has a small distal lobe of A9s fused with the mesoventral edge of the gonocoxite (ref. [43]: Figures 26 and 27; ref. [44]: Figure 7) with a shape similar to the mesoventral lobe of the lateral sclerite of the gonocoxal fragment. The fusion with gonocoxite, shape, and position of both sclerites attribute to possible homology. Still, additional comparative studies, including muscle morphology, are needed to prove the origin of the distal lobe of A9s.

According to Savchenko [45] the genus *Brithura* is closely related to *T*. (*Nippotipula*) and *T*. (*Sinotipula*) Alexander, 1935 (listed as *T*. (*Bellardina*) Edwards, 1931) based on several external features including hypertrophic. outer gonostylus. Alexander noted the complexity of the outer gonostylus in *T*. (*Nippotipula*), *T*. (*Sinotipula*) and *T*. (*Bellardina*), which is small and simple in other groups [46]. All mentioned groups, together with *T*. (*Nobilotipula*) Alexander, 1943 form a separate clade based on AHE dataset, which includes 289 taxa and 257 loci (ref. [47]: Figure 4). Based on *COI* sequences *T*. (*Nippotipula*) did not come out close to *T*. (*Bellardina*) or *Brithura* (ref. [47]: Figure 1).

Currently it is difficult to make decisive conclusions on correlations between morphological and genetic species comparisons. Unfortunately, *COI* gene sequences of possibly very closely related subgenera of *T. (Sinotipula)* and *T. (Bellardina)* are not available on databases. In general, depending on the genus, crane fly intraspecific genetic variability of *COI* sequences is somewhere above ~94% nucleotide identity, while interspecific similarities are below this mark. This was also the case with our *B. sancta* samples. However, usually quite a few different genera share very similar nucleotide sequence identity when comparing each one to another. This makes it difficult to distinguish their exact genetic connections and phylogeny trees are generated with low bootstrap values on branches between genera (Figure 13). Notably, a relatively short, ~650 bp size *COI* gene fragment is used globally for insect identification and classification, and full gene length ~1500 bp sequences are absent. While this fragment is usually used for identification of species, it alone might be not sufficient to clearly and fully distinguish genera relations and connections in such a large family as Tipulidae (over thousands [1]) and additional genetic markers might be needed for this purpose.

Larvae of the genus *Brithura* exhibit the greatest morphological similarity to those of *Tipula* from subgenera *T*. (*Acutipula*) Alexander, 1924 and *T*. (*Tipula*). These larvae are notably large and lack lobes or other projections on the abdominal segments. The body surface in *Brithura*, as well as in *T.* (*Acutipula*) and *T.* (*Tipula*), is covered only with fine, microscopic setae; conspicuous macrosetae forming longitudinal or transverse patterns are absent.

Like the majority of aquatic and semi-aquatic Tipulidae larvae, *Brithura* possesses a full complement of macrosetae (D1–D6, V1–V5, L1–L4). The size, shape, and arrangement of these macrosetae are diagnostic at the genus or subgenus level within Tipulidae [11]. Certain features—such as branched macrosetae D6, L3, V1, and the presence of particularly long setae—are shared among several aquatic and semi-aquatic groups, including *Angarotipula*, *Tipulodina*, *T.* (*Arctotipula*) Alexander, 1934, *T.* (*Platytipula*) Matsumura, 1916, *T.* (*Bellardina*), *T.* (*Nobilotipula*) Alexander, 1943, and *T.* (*Yamatotipula*) Matsumura, 1916. In *Brithura*, setae V2 and D5 are surrounded by clusters of longer microsetae, though these are much shorter than in *T.* (*Yamatotipula*). While the general arrangement of dorsal, lateral, and ventral setae resembles that of *T.* (*Sinotipula*), branched setae are not characteristic of the latter [35].

The larval head capsule is highly conserved across Tipulidae and is generally not useful for genus-level identification [36]. Moreover, descriptions of tipulid larvae often provide only superficial or incomplete accounts of head capsule morphology, complicating comparative diagnoses. In this family, the hypostomal plate bears a variable number of teeth, typically ranging from 5 to 11 [25,35]. *Brithura*, like *Ctenophora*, *Phoroctenia*, *Tanyptera*, *Prionocera*, *Tipulodina*, and the subgenera *T*. (*Dendrotipula*) Savchenko, 1964 and parts of *T*. (*Pterelachisus*) Rondani, 1842, possesses nine hypostomal teeth.

The number of anal papillae is a key taxonomic feature in Tipulidae larvae [6,35]. In *Brithura*, the anus is surrounded by three pairs of elongated anal papillae—a common number among aquatic and semi-aquatic genera, including *Angarotipula*, *Prionocera*, and the subgenera *T*. (*Platytipula*), *T*. (*Nippotipula*), *T*. (*Arctotipula*), *T*. (*Sinotipula*), and *T*. (*Yamatotipula*) of genus *Tipula*.

Among the most reliable diagnostic characters for Tipulidae larvae is the architecture of the spiracular field. All known Tipulidae larvae possess six spiracular lobes, with lobe shape varying according to habitat type and taxonomical group. Aquatic species typically have flat or conical lobes that are only weakly sclerotized. *Brithura*, like *T.* (*Nippotipula*) and *T.* (*Arctotipula*), exhibits long, conical spiracular lobes. However, in contrast to the latter subgenera, the lobes in *Brithura* are more uniform in length and less differentiated. A distinguishing feature of *Brithura* is the presence of relatively long setae along the margins of the spiracular lobes—a character which is absent in *T.* (*Nippotipula*) and *T.* (*Arctotipula*). Additionally, the dorsal surfaces of the ventral and lateral lobes bear weakly developed setose outgrowths. Although similar outgrowths occur in *T.* (*Nippotipula*), they are more pronounced and represent a major diagnostic feature of that subgenus. The most distinguishing character of *Brithura* is the unique sclerotisation of the spiracular field. The unique set of characters of the spiracular field in *Brithura* larvae reinforces the consensus that this structure provides the most consistent and informative characters for distinguishing genera and subgenera in Tipulidae larvae.

The larval habitat of *Brithura* closely resembles that of *T.* (*Nippotipula*), *T.* (*Arctotipula*), and certain species within *T.* (*Acutipula*). Larvae of all these groups develop in lotic environments, typically on the beds of small streams with sandy or stony substrates, where fine organic silt accumulates among the interstitial spaces.

## Figures and Tables

**Table 1:** List of genera of Tipulidae with known larvae.

Genus	Link to Reference Having First Illustrations
*Angarotipula* Savchenko, 1961	[9]
*Brachypremna* Osten Sacken, 1887	[10]
*Ctenophora* Meigen, 1803	[11]
*Dictenidia* Brulle, 1833	[11]
*Dolichopeza* Curtis, 1825	[11]
*Holorusia* Loew, 1863	[9]
*Indotipula* Edwards, 1931	[12]
*Leptotarsus* Guerin-Meneville, 1831	[13]
*Maekistocera* Wiedemann, 1820	[14]
*Nephrotoma* Meigen, 1803	[11]
*Nigrotipula* Hutson and Vane-Wright, 1969	[15]
*Phoroctenia* Coquillett, 1910	[16]
*Prionocera* Loew, 1844	[11]
*Ptilogyna* Westwood, 1835	[17]
*Tanyptera* Latreille, 1804	[11]
*Tipula* Linnaeus, 1758	[18]
*Tipulodina* Enderlein, 1912	[19]
*Zelandotipula* Alexander, 1922	[20]

**Table 2:** Information for voucher specimens used in *COI* barcode analyses.

Accession/Specimen Number	Database	Species
OQ368738 *	GenBank	*Brithura sancta* Alexander, 1929
OQ368739 *	GenBank	*Brithura sancta* Alexander, 1929
OQ368740 *	GenBank	*Brithura sancta* Alexander, 1929
PV834789 *	GenBank	*Brithura sancta* Alexander, 1929
ON732830	GenBank	*Brithura* sp.
ON732831	GenBank	*Brithura* sp.
ON732832	GenBank	*Brithura* sp.
ON732833	GenBank	*Brithura* sp.
ON732834	GenBank	*Brithura* sp.
ON732835	GenBank	*Brithura* sp.
CMNH544017	BOLD	*Brithura* sp.
CMNH544493	BOLD	*Brithura* sp.
CMNH544500	BOLD	*Brithura* sp.
CMNH551721	BOLD	*Brithura* sp.
CMNH575752	BOLD	*Brithura* sp.
CMNH544017	BOLD	*Brithura* sp.
ZFMK-TIS-2004136	BOLD	*Tipula* (*Acutipula*) *luna* Westhoff, 1879
CHARS00030-D06	BOLD	*Tipula* (*Arctotipula*) *salicetorum* Siebke, 1870
BIOUG00728-B09	BOLD	*Tipula* (*Beringotipula*) *coloradensis* Doane, 1911
CMNH544749	BOLD	*Tipula* (*Beringotipula*) *comstockiana* Alexander, 1947
CMNH407584	BOLD	*Tipula* (*Nippotipula*) *abdominalis* (Say, 1823)
CMNH544543	BOLD	*Tipula* (*Nippotipula*) *coquilletti* Enderlein, 1912
CMNH475020	BOLD	*Tipula* (*Nobilotipula*) *collaris* Say, 1823
CARR008A5	BOLD	*Tipula* (*Schummelia) variicornis* Schummel, 1833
INV09325	BOLD	*Tipula* (*Schummelia*) *yerburyi* Edwards, 1924
INV04294	BOLD	*Tipula* (*Tipula*) *mediterranea* Lackschewitz, 1930
BIOUG01427-G01	BOLD	*Tipula* (*Tipula*) *oleracea* Linnaeus, 1758
BIOUG56888-H10	BOLD	*Nephrotoma altissima* (Osten Sacken, 1877)
CNCDIPTERA106679	BOLD	*Phalacrocera replicata* (Linnaeus, 1758)

*—sequences of *COI* fragment obtained in this study.

## Data Availability

The original contributions presented in this study are included in the article. Further inquiries can be directed to the corresponding author.
